# On a new species of freshwater crab, *Indochinamonkhinpyae*, from northern Myanmar (Crustacea, Brachyura, Potamidae)

**DOI:** 10.3897/zookeys.811.29187

**Published:** 2018-12-31

**Authors:** Peter K. L. Ng, Win Mar

**Affiliations:** 1 Lee Kong Chian Natural History Museum, 2 Conservatory Drive, National University of Singapore, Singapore 117377, Republic of Singapore National University of Singapore Singapore Singapore; 2 Department of Zoology, Banmaw University, Banmaw, Kachin State, Republic of the Union of Myanmar Banmaw University Banmaw Myanmar

**Keywords:** Taxonomy, freshwater crab, Burma, Potaminae, *
Indochinamon
*, new species, description

## Abstract

A new species of freshwater crab of the genus *Indochinamon* Yeo & Ng, 2007 (family Potamidae), is described from highlands north of Myitkyina in Kachin State, Myanmar. *Indochinamonkhinpyae***sp. n.** is distinguished from congeners by its very rugose carapace, broad male pleon and distinctively structured male first gonopod; and is the first potamid species recorded from northern Myanmar.

## Introduction

The freshwater crabs (Brachyura, Potamidae, Gecarcinucidae) of Indochina are very diverse, with the fauna still in a survey and discovery stage. The fauna of Myanmar (= Burma) in particular, is poorly known, with most of the recognised species described in the early 1900s (see Yeo and Ng 1999; Cumberlidge et al. 2012). Recent efforts in recording the crab fauna have only just started (e.g., Ng 1996, 2018; Ng and Kosuge 1997; Ng and Whitten 2017), with many parts of the country still barely explored. The second author recently obtained several lots of freshwater crabs from northern Myanmar, one of which proved to belong to a new species of Potamidae.

## Material and methods

The terminology used follows Ng (1988) with recent changes by Davie et al. (2015). The abbreviations G1 and G2 are used for the male first and second gonopods, respectively. Measurements provided, in millimetres, are of the maximum carapace width and length, respectively. The material examined is deposited in the Museo Civico di Storia Naturale “Giacoma Doria” (MGE), Genova, Italy; Naturhistorisches Museum Basel (MBA), Basel, Switzerland; Muséum national d’Histoire naturelle (MNHN), Paris, France; Naturalis [formerly Rijksmuseum van Natuurlijke Histoire, RMNH], Leiden, The Netherlands; Senckenbergischen Naturforschenden Gesellschaft (SMF), Frankfurt am Main, Germany; Zoological Reference Collection (ZRC), Lee Kong Chian Natural History Museum, National University of Singapore; and the Zoological Survey of India (ZSI), Calcutta, India.

## Systematics

### Family Potamidae Ortmann, 1896 sensu Yeo and Ng (2004)

#### Genus Indochinamon

Taxon classificationAnimaliaDecapodaPotamidae

Yeo & Ng, 2007

##### Type species.

*Potamonvillosum* Yeo & Ng, 1998, by original designation.

##### Remarks.

The genus currently contains 38 species from Thailand, Vietnam, Laos, Myanmar, India and China (Table 1, updated from Ng et al. 2008; Naruse et al. 2018). Established by Yeo and Ng (2007) for Indochinese species previously placed in *Potamon* Savigny, 1816, s. lato, *Indochinamon* is defined by a suite of characters: carapace low with a relatively flat dorsal surface; the epigastric cristae are separated from the postorbital cristae by a distinct groove; the postorbital cristae is not confluent with the epibranchial tooth; the exopod of the third maxilliped has a long flagellum; the ambulatory legs are relatively short and stout; the male pleon is narrowly triangular; the sternopleonal cavity reaches an imaginary line joining the median parts of the coxae of the chelipeds; and the G1 terminal segment is relatively short, with the groove for the G2 marginal in position, and the dorsal flap is either absent or only low and broad.

**Table 1.** List of recognised *Indochinamon* species.

*Indochinamonahkense* Naruse, Chia & Zhou, 2018 [type locality: Guangnan County, Yunnan Province, China]
*Indochinamonandersonianum* (Wood-Mason, 1871) [type locality: Momein, west Yunnan Province, China]
*Indochinamonasperatum* (Alcock, 1909) [type locality: Cachar Hills, India]
*Indochinamonbavi* Naruse, Nguyen & Yeo, 2011 [type locality: Ha Tay Province, northern Vietnam]
*Indochinamonbeieri* (Pretzmann, 1966) [type locality: Dawane Hills, India]
*Indochinamonbhumibol* (Naiyanetr, 2001) [type locality: Loei Province, northern Thailand]
*Indochinamonboshanense* (Dai & Chen, 1985) [type locality: Boshan, Yunnan Province, China]
*Indochinamonchangpoense* (Dai, 1995) [type locality: Jingping, Yunnan Province, China]
*Indochinamonchinghungense* (Dai, Song, He, Cao, Xu & Zhong, 1975) [type locality: Ching Hung, Yunnan Province, China]
*Indochinamonchuahuong* Do, Nguyen & Le, 2016 [type locality: Ha Noi province, northern Vietnam]
*Indochinamoncua* (Yeo & Ng, 1998) [type locality: Vinh Phu Province, northern Vietnam]
*Indochinamondangi* Naruse, Nguyen & Yeo, 2011 [type locality: Dien Bien Province, northern Vietnam]
*Indochinamondaweishanense* (Dai, 1995) [type locality: Daweishan, Yunnan Province, China]
*Indochinamonedwardsii* (Wood-Mason, 1871) [type locality: Ponsee, Kahkyen Hills, Yunnan Province, China]
*Indochinamonflexum* (Dai, Song, Li & Liang, 1980) [type locality: Napo, Guangxi Province, China]
*Indochinamongengmaense* (Dai, 1995) [type locality: Gengma, Yunnan Province, China]
*Indochinamonguttum* (Yeo & Ng, 1998) [type locality: Muang Saisombun, northern Laos]
*Indochinamonhirtum* (Alcock, 1909) [type locality: Sheetee Hills, Kakhyen Hills, Yunnan Province, China]
*Indochinamonhispidum* (Wood-Mason, 1871) [type locality: Ponsee, Kakhyen Hills, Yunnan Province, China]
*Indochinamonjianchuanense* (Dai & Chen, 1985) [type locality: Hengduan, Yunnan Province, China]
*Indochinamonjinpingense* (Dai, 1995) [type locality: Yunnan Province, China]
*Indochinamonkhinpyae* sp. n. [type locality: Kachin State, Myanmar]
*Indochinamonkimboiense* (Dang, 1975) [type locality: Kim Boi Province, northern Vietnam]
*Indochinamonlipkei* (Ng & Naiyanetr, 1993) [type locality: Chiang Rai Province, northern Thailand]
*Indochinamonlui* Naruse, Chia & Zhou, 2018 [type locality: Yun County, Yunnan Province, China]
*Indochinamonmanipurense* (Alcock, 1909) [type locality: Manipur Hills, India]
*Indochinamonmenglaense* (Dai & Cai, 1998) [type locality: Xishuangbana, Yunnan Province, China]
*Indochinamonmieni* (Dang, 1967) [type locality: Son La Province, northern Vietnam]
*Indochinamonorleansi* (Rathbun, 1904) [type locality: northern Vietnam]
*Indochinamonou* (Yeo & Ng, 1998) [type locality: Phongsali Province, northern Laos]
*Indochinamonparpidum* Naruse, Chia & Zhou, 2018 [type locality: Shiping County, Yunnan Province, China]
*Indochinamonphongnha* Naruse, Nguyen & Yeo, 2011 [type locality: Quang Binh Province, central Vietnam]
*Indochinamonprolatum* (Brandis, 2000) [type locality: Uthai Thani Province, central Thailand]
*Indochinamontannanti* (Rathbun, 1904) [type locality: Lao Koi, Yunnan Province, China]
= *Potamonhokuoense* Dai, Song, He, Cao, Xu & Zhong, 1975 [type locality: Hekou, Yunnan Province, China]
*Indochinamontritum* (Alcock, 1909) [type locality: Sheetee Hills, Kakhyen Hills, Yunnan Province, China]
*Indochinamontujiense* Naruse, Chia & Zhou, 2018 [type localiy: Nanhua County, Yunnan Province, China]
*Indochinamonvillosum* (Yeo & Ng, 1998) [type locality: Luang Nam Tha Province, northern Laos]
*Indochinamonxinpingense* (Dai & Bo, 1994) [type locality: Yuxi, Yunnan Province, China]
= *Potamonhispidumxingpingense* Bo, He, Huang, Fan, Dai & Chen, 1997 [type locality: Yuxi, Yunnan Province, China]
*Indochinamonyunlongense* (Dai, 1995) [type locality: Yunlong, Yunnan Province, China]

#### Indochinamon khinpyae sp. n.

Taxon classificationAnimaliaDecapodaPotamidae

http://zoobank.org/878C53F6-38DA-42C3-B282-A1F6A56C0E92

Figs 1 , 2 , 3 , 4

##### Material examined.

Holotype: male (57.1×43.2 mm) (ZRC 2018.0713), Malikha River, about 3.2 km from confluence point of Ayeyarwady River, north of Myitkyina, Kachin State, Myanmar, coll. Khin Pyae Pyae Thaw Thar, May 2018. Paratypes: 5 males (47.2×36.4 mm, 48.9×37.7 mm, 46.4×35.8 mm, 34.3×26.6 mm, 26.7×21.1 mm), 1 female (39.3×31.1 mm) (ZRC 2018.0714), same data as holotype.

**Figure 1.**
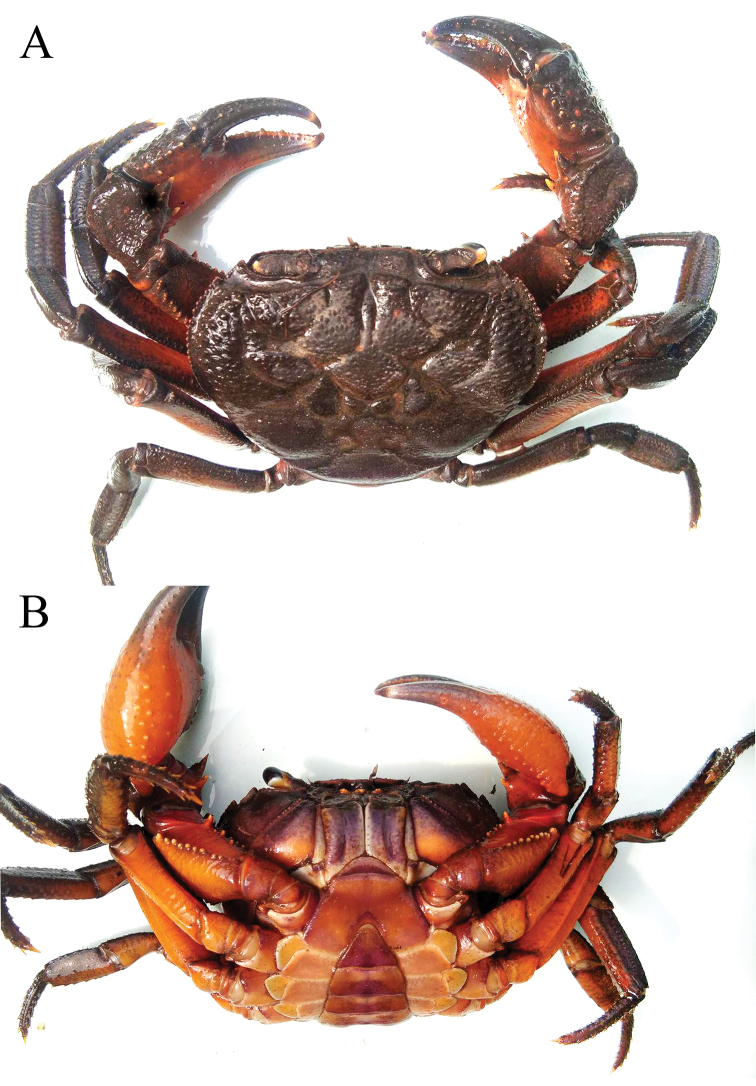
*Indochinamonkhinpyae* sp. n., colour in life, holotype male (57.1×43.2 mm) (ZRC 2018.0713). **A** dorsal view **B** ventral view of cephalothorax.

##### Comparative material.

*Indochinamonahkense* Naruse, Chia & Zhou, 2018 – paratypes: 4 males (largest 38.4×29.7 mm), 2 females (larger 43.1×33.2 mm) (ZRC 2013.0551), Shaping Village, Ahke Town, Guangnan County, Yunnan Province, China, coll. Z.L. Chen, 1 February 2004. *Indochinamonandersonianum* (Wood-Mason, 1871) – syntypes: 2 males (larger 36.4×28.3 mm), 1 female (42.9×33.5 mm) (ZSI 4045/4), Momein, West Yunnan, China, coll. J. Wood-Mason, no date; 1 male (49.5×37.0 mm) (SMF 2805), Mt. Carien, Myanmar, coll. L. Fea, 1885–1889; 1 female (47.9×36.9 mm) (ZSI 6916/3), Yunnan, coll. J. Anderson, no date; 11 juveniles (ZS1 6932/3), West Yunnan and Kahkhyen Hills, coll. J. Anderson, no date; 1 juvenile male, 3 females (largest 33.8×26.8 mm) (ZSI 6906/3), Kahkhyen Hills, Ponsee, Upper Burma, coll. J. Anderson, no date. *Indochinamonasperatum* (Alcock, 1909) – syntypes: 4 juvenile males (largest 18.1×15.3 mm), 1 juvenile female (19.2×15.7 mm) (ZSI 5543/10), Ganjam, Cachar Hills, coll. W. Partridge, no date. *Indochinamonbavi* Naruse, Nguyen & Yeo, 2011 – paratypes: 2 males (46.9×35.8 mm, 47.5×36.3 mm) (ZRC 2010.0167), Ba Vi National Park, Ha Tay Province, Vietnam, coll. V.Q. Nguyen, 19 June 2001. *Indochinamonbhumibol* (Naiyanetr, 2001) – 3 males (ZRC), Ban Nam Tob, Khao Luang, Amphoe Wang Saphung, Loei Province, Thailand, coll. W. Senama, 26 October 1982; 1 male (ZRC), Huai Phai Waterfall, Phu Rua, Amphoe Phu Rua, Loei Province, coll. Wiroon, 24 July 1982; 2 males (ZRC), Ban Na Wa, Amphoe Dan Sai, Loei Province, Thailand, coll. P. Naiyanetr, 11 April 1987. *Indochinamonboshanense* (Dai & Chen, 1985) – 1 male (50.4×37.1 mm) (ZRC 1998.811), Boshan, Yunnan Province, China, coll. A. Dai, 20 October 1981. *Indochinamonchinghungense* (Dai, Song, He, Cao, Xu & Zhong, 1975) – 1 male (50.5×38.2 mm) (ZRC 1997.749), Menghai County, 100 m asl, Yunnan County, China, coll. Y. Cai, 11 May 1994. *Indochinamoncua* (Yeo & Ng, 1998) – holotype: male (46.9×36.6 mm) (ZRC 1998.267), Tam Dao, Vinh Phu Province, northern Vietnam, coll. X.Q. Nguyen, 8 June 1997; paratypes: 2 females (larger 42.6×33.0 mm), 2 juvenile males (ZRC 1998.268–271), same locality and collector as holotype, March 1997. *Indochinamondangi* Naruse, Nguyen & Yeo, 2011 – 4 males (29.6×23.0 – 48.4×37.6 mm), 2 females (45.2×33.9, 36.7×28.1 mm) (ZRC 2010.0175), upstream and waterfall of Muong Phang stream, Muong Phang, Dien Bien Province, 21°27.000'N, 103°10.548'E, 1070 m asl, coll. D.C.J. Yeo and A.D. Tran, 28 July 2004; 4 males (26.7×20.7 – 41.8×32.2 mm), 1 female (46.7×35.5 mm) (ZRC 2010.0176), Muong Phang stream, Muong Phang, Dien Bien Province, 21°27.159'N, 103°09.921'E, 976m asl, coll. D.C.J. Yeo and A.D. Tran, 26 July 2004. *Indochinamonedwardsii* (Wood-Mason, 1871) – 1 male (40.9×30.3 mm), 1 female (37.5×27.5 mm) (MGE III 228 bis), Mt. Catcin, Birmania (= Myanmar), coll. L. Fea, June–October 1886; 1 male (38.5×28.9 mm), 2 females (larger 33.9×25.5 mm) (MBA 51a), Katein Berge, northern Burma; 1 male (about 34.4 mm carapace width) (ZRC 1984.7036), Mount Katun, Burma (= Myanmar), coll. L. Fea, 1893. *Indochinamonflexum* (Dai, Song, Li & Liang, 1980) – 1 male (47.5×35.0 mm) (ZRC 1997.0750), Guangxi Autononmous Region, China, coll. Y. Song, 16 September 1997. *Indochinamonguttum* (Yeo & Ng, 1998) – holotype: male (62.2×45.7 mm) (ZRC 1998.272), Ban Long Cheng, Muang Saisombun, Saisombun Special Zone, northern Laos, coll. V. Kittikoon, May 1995; paratypes: 1 male (43.8×34.7 mm) (ZRC 1998.273), same data as holotype; 1 female (33.1×25.3 mm) (ZRC 1998.0274), side of dam, Muang Saisombun, Saisombun Special Zone, northern Laos, coll. V. Kittikoon, May 1995; 8 specimens (largest male 51.3×38.8 mm) (MNHN-B 5316) “Haut Laos, Ban Nong”, coll. Mission Permanente, 10 January 1906. *Indochinamonhirtum* (Alcock, 1909) – holotype: female (32.9×24.9 mm) (ZSI 6961/3), Sheetee Hill (= Shitee Doung), Kakhyen Hills, Yunnan, China, coll. J. Anderson, no date; 5 males (largest 37.8×28.0 mm), 2 females (ZSI 6961/3), same data as holotype. *Indochinamonhispidum* (Wood-Mason, 1871) – 1 juvenile female (ZSI 4007/4), Kakhyen Hills, Ponsee, Upper Burma, coll. J. Anderson, no date; 1 female (34.6×26.9 mm), 1 juvenile male (ZSI 7089-90/9), Moung Sal, Mehkok River, coll. Dr. Grey, no date. *Indochinamonjinpingense* (Dai, 1995) – 2 males (larger 64.0×47.0 mm) (ZRC 1998.266), Sin Ho District, Lai Chau Province, northern Vietnam, coll. V.D. Nguyen, November 1997. *Indochinamonkimboiense* (Dang, 1975) – 2 males (71.8×56.6 mm, 71.5×56.8 mm), 2 females (63.0×49.5 mm, 52.8×41.1 mm) (ZRC 2010.0165), Kim Boi area, Hoa Binh Province, Vietnam, purchased from villagers, 14 and 15 April 2007; 1 male (58.8×45.3 mm), 2 females (69.4×53.6 mm, 49.9×37.4 mm) (ZRC 2010.0166), stream in Cuc Phuong National Park, about 6 km from main gate, Ninh Binh Province, northern Vietnam, 20°18'N, 105°38'E, coll. D.C.J. Yeo, H.H. Ng and X.Q. Nguyen, 16 September 1997. *Indochinamonlipkei* (Ng & Naiyanetr, 1993) – holotype: male (56.8×42.8 mm) (RMNH D 42353), Chiang Khong District, Chiang Rai Province, northwestern Thailand, coll. P. Naiyanetr, June 1987. *Indochinamonmanipurense* (Alcock, 1909) – syntypes: 1 male (39.9×31.5 mm), 1 female (40.1×30.4 mm) (ZSI 6923/3), Manipur Hills, India, coll H. H. Godwin-Austen, no date. *Indochinamonmenglaense* (Dai & Cai, 1998) – 1 male (42.9×31.9 mm), 1 female (ZRC), Shangyong, Xishuangbana, Yunnan, China, coll. Y. Cai, 23 April 1994. *Indochinamonmieni* (Dang, 1967) – neotype: male (57.1×43.5 mm) (ZRC 1998.265), Thuan Chau District, Son La Province, northern Vietnam, coll. V.D. Nguyen, 1997; 1 juvenile female (ZRC), same data as neotype. *Indochinamonorleansi* (Rathbun, 1904) – holotype: male (42.4×32.3 mm) (MNHN-B 5262), “Tonkin, rivière Noire” (river Song Da), coll. Prince Henri d’Orleans, no date. *Indochinamonou* (Yeo & Ng, 1998) – holotype: male (35.6×27.1 mm) (ZRC 1998.275), Nam Ou at confluence with Huay Nam, 21°4'10"N, 102°31'44"E, 3 km ESE of Muang Khoa, Phongsali Province, northern Laos, coll. M. Kottelat, 17 May 1997; 1 male (47.6×36.8 mm) (ZRC), dry evergreen forest mixed with bamboo, Nam Sa River, tributary of Nam Ou, 600 m asl, 22°5'31"N, 102°6'19"E, Phou Dendin, Phonsgali, northern Laos, coll. and date not known. *Indochinamonparpidum* Naruse, Chia & Zhou, 2018 – paratypes: 2 males (larger 43.4×32.3 mm), 2 females (larger 35.0×26.2 mm) (ZRC 2013.0558), Niujie Town, Shiping County, Yunnan Province, China, coll. H.C. Li, 23 February 2004. *Indochinamonphongnha* Naruse, Nguyen & Yeo, 2011 – paratypes: 5 males (25.4×20.2 – 44.2×33.2 mm), 6 females (17.9×14.1 – 43.0×32.6 mm), 1 juvenile (15.4×12.5 mm) (ZRC 2010.0168), Khe Con Khai stream, Cha Noi, Phong Nha, Quang Binh Province, Vietnam,17°38.196'N, 106°05.928'E, 263 m asl, coll. D.C.J. Yeo and A.D. Tran, 13 July 2004; 2 males (34.3×26.6 mm, 31.9×24.9 mm), 3 females (38.3×29.9 – 54.8×41.4 mm), 1 juvenile (17.0×13.1 mm) (ZRC 2010.0169), Cha Noi, Phong Nha, Quang Binh Province, Vietnam, Stream under bridge, 17°38.397'N, 106°06.975'E, 261 m asl, coll. D.C.J. Yeo and A.D. Tran, 13 July 2004; 11 males (16.4×13.3 – 64.2×48.0 mm), 3 females (32.6×25.9 – 38.9×30.1 mm) (ZRC 2010.0170), Vuc Tro stream, Phong Nha, Quang Binh Province, 17°38.188'N, 106°12.810'E, coll. D.C.J. Yeo and A.D. Tran, 14 July 2004; 3 females (36.1×28.1 – 42.8×33.0 mm), 2 juveniles (19.6×15.3 mm, 17.3×13.6 mm) (ZRC 2010.0171), stream near Forest Ranger station 37, Phong Nha, Quang Binh Province Vietnam, 17°31.395'N, 106°17.716'E, 86 m asl, coll. D. C. J. Yeo and A. D. Tran, 15 July 2004; 3 males (49.8×37.8 – 53.0×41.5 mm) (ZRC 2010.0172), Chay stream, Quang Binh Province, Vietnam, 17°33.146'N, 106°14.425'E, 94 m asl, coll. D.C.J. Yeo and A.D. Tran, 17 July 2004; 1 male (61.9×47.3 mm) (ZRC 2010.0173), Km 23 + 800 HCM Way, near Hang So Dua, Pong Nha National Park, Quang Binh Province, Vietnam, coll. A.D. Tran, 11 August 2001; 1 male (56.9×44.0 mm), 1 female (54.2×40.8 mm) (ZRC 2010.0174), Thac Xoi waterfall, Phong Nha National Park, Quang Binh Province, Vietnam, coll. Q.K. Hoang and V.K. Dinl, 10 August 2002. *Indochinamontannanti* (Rathbun, 1904) – holotype: female (35.5×27.7 mm) (MNHN-B 5313), “Tonkin, montagnes du Yunnan (via Lao Koi)”, coll. Tannant, no date; 1 male (56.1×42.6 mm) (ZRC 1998.264), Hekou, Yunnan Province, southern China, coll. A.-Y. Dai, 29 November 1995. *Indochinamontritum* (Alcock, 1909) – holotype: female (35.8×27.4 mm) (ZSI 4075/4), Sheetee Hill (= Shitee Doung), Kakhyen Hills, Yunnan, China, coll. J. Anderson, no date. *Indochinamontujiense* Naruse, Chia & Zhou, 2018 – paratypes: 2 males (larger 33.1×25.0 mm), 1 female (31.4×23.6 mm) (ZRC 2013.0555), Mang Huai Town, Yun County, Yunnan Province, China, coll. Y.F. Lu, 24 February 2004; 2 males (larger 37.5×28.8 mm), 2 females (larger 38.6×28.6 mm) (ZRC 2013.0557), Mongku Town, Shuangjiang County, Yunnan Province, China, coll. O.C. Li, 26 February 2004. *Indochinamonvillosum* (Yeo & Ng, 1998) – holotype: male (44.8×34.3 mm) (ZRC 1998.276), tributary of Nam Tha River about 800 m asl, Luang Nam Tha Province, northern Laos, coll. H. Morioka, 13 November 1997; paratypes: 7 males (largest 55.9×41.4 mm), 4 females, 1 juvenile (ZRC 1998.277–285, 807–809), same data as holotype; 2 females (larger 32.9×24.5 mm) (ZRC 1998.286–287), Nam Luang about 1 km upstream of Ban Nam Luang, Nam Tha watershed, Mekong basin, Luang Nam Tha Province, northern Laos, 21°9'5"N, 101°20'34"E, coll. M. Kottelat, 22 May 1997; 1 female (39.9×30.8 mm) (ZRC 1998.288), tributary of Nam Talan about 3 km S of Ban Nateuy, Nam Tha watershed, Mekong basin, Luang Nam Tha Province, northern Laos, coll. M. Kottelat, 20 May 1997.

##### Diagnosis.

**Figure 2.**
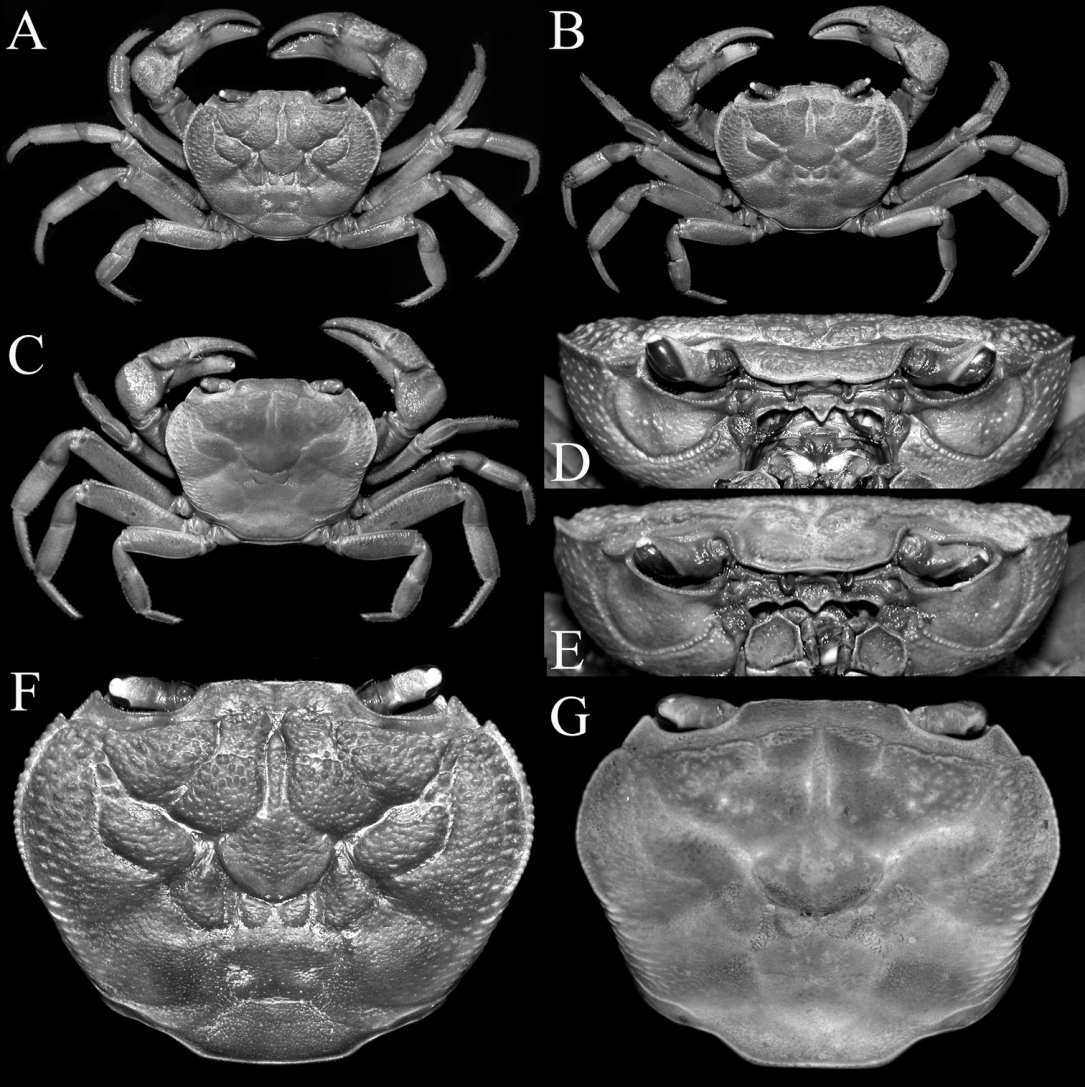
*Indochinamonkhinpyae* sp. n. **A, D, F** holotype male (57.1×43.2 mm) (ZRC 2018.0713) **B, E** paratype male (47.2×36.4 mm) (ZRC 2018.0714) **C, G** paratype male (34.3×26.6 mm) (ZRC 2018.0714). **A–C** overall habitus **D, E** frontal view of cephalothorax **F, G** dorsal view of carapace.

**Figure 3.**
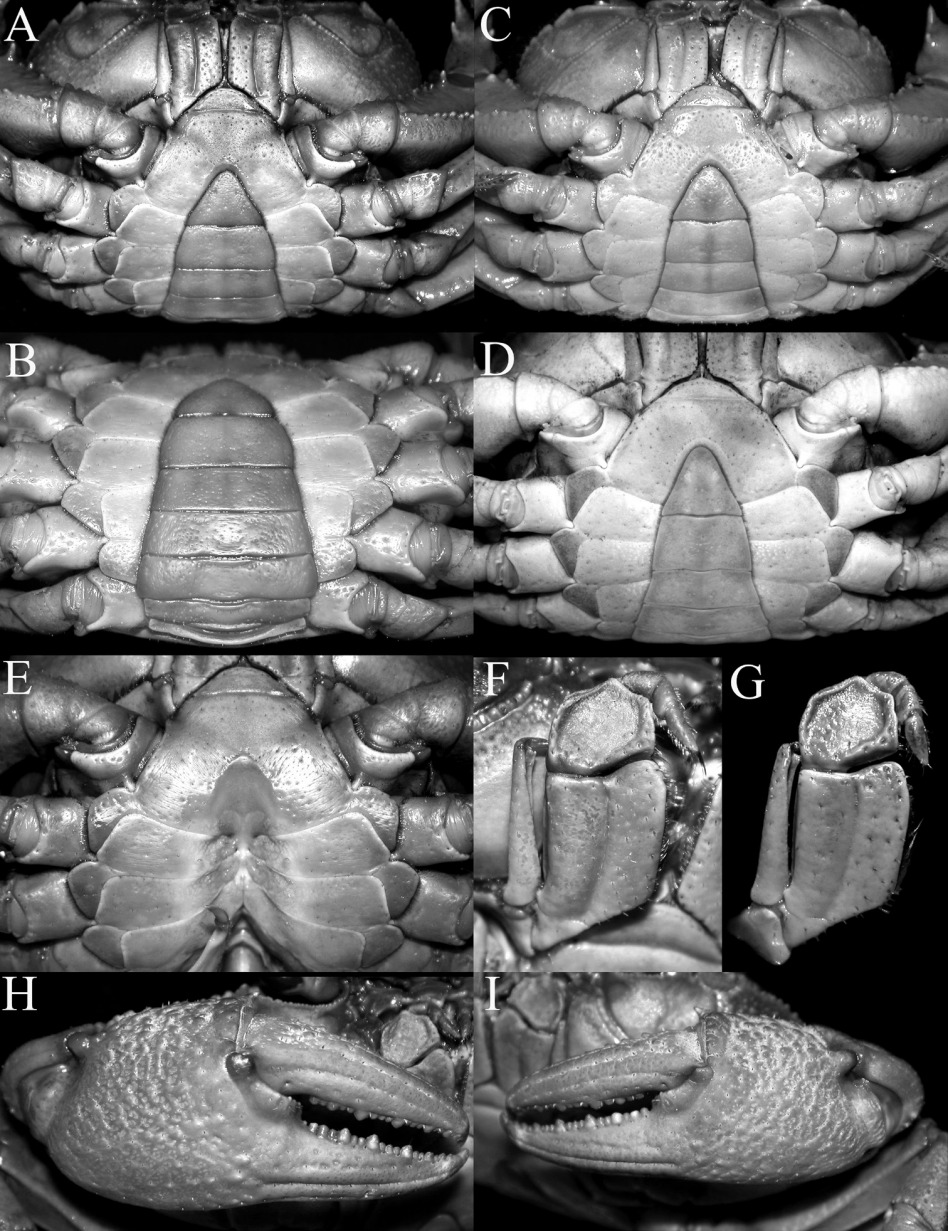
*Indochinamonkhinpyae* sp. n. **A, B, E, F, H, I** holotype male (57.1×43.2 mm) (ZRC 2018.0713) **C, G** paratype male (47.2×36.4 mm) (ZRC 2018.0714) **D** paratype male (34.3×26.6 mm) (ZRC 2018.0714). **A, C, D** anterior thoracic sternum and pleon **B** posterior thoracic sternum and pleon **E** sternopleonal cavity **F, G** right third maxilliped **H** outer view of right chela **I** outer view of left chela.

**Figure 4.**
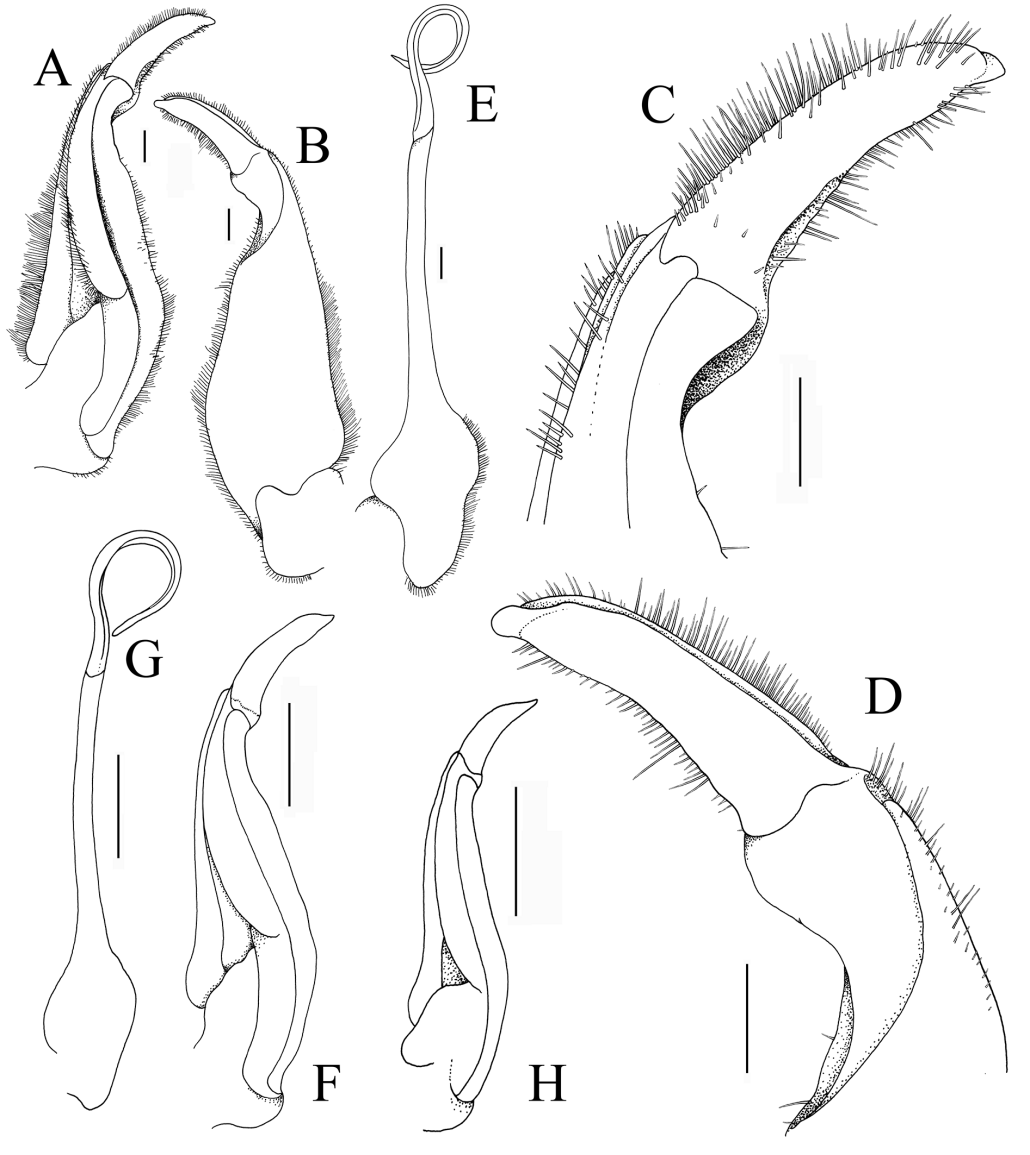
*Indochinamonkhinpyae* sp. n. **A–E** holotype male (57.1×43.2 mm) (ZRC 2018.0713); **F, G** paratype male (47.2×36.4 mm) (ZRC 2018.0714) **H** paratype male (34.3×26.6 mm) (ZRC 2018.0714). **A** left G1 (ventral view) **B** left G1 (dorsal view) **C** terminal segment of left G1 (ventral view) **D** terminal segment of left G1 (dorsal view) **E** left G2 **F, H** left G1 (ventral view, setae not drawn) **G** left G2 (setae not drawn). Scale bar: 1.0 mm.

Carapace with dorsal surface prominently rugose in large specimens (ca. 45 mm carapace width), frontal and orbital regions prominently rugose, lateral parts of anterolateral and branchial regions with strong oblique striae; mesogastric, urogastric, cardiac and intestinal regions with distinct rugosities and distinct granules (Fig. 2A, D, F); postorbital cristae distinct, margin uneven, outer edge relatively low, not well marked (Fig. 2A, D, F); external orbital tooth distinct, separated from anterolateral margin by deep V-shaped cleft; epibranchial tooth prominent (Fig. 2A, F); anterolateral margin lined with sharp granules, appears serrated (Fig. 2A, F); posterior margin of epistome with distinct median triangle (Fig. 2D); outer surface of chela strongly rugose, upper part rugose with granules (Fig. 3H, I); male thoracic sternum, notably sternites 3 and 4, relatively broad, surface with pits and scattered short, stiff setae (Fig. 3A); male pleon triangular; telson triangular, lateral margins gently sinuous; somite 6 transversely rectangular, much wider than long, lateral margin gently convex (Fig. 3A, B); G1 relatively stout; outer margin of subterminal segment with distinct broad cleft on distal part, terminal segment subcylindrical, gently curving outwards, no visible dorsal flap, distal part tapering to rounded tip (Fig. 4A–D).

##### Description of male holotype.

Carapace transversely ovate, distinctly wider than long (width to length ratio 1.32); dorsal surface gently convex from frontal view, regions not prominently inflated; with scattered very short setae, appears glabrous (Fig. 2A, D, F). Frontal and orbital regions prominently rugose; lateral parts of anterolateral and branchial regions covered with strong oblique striae; mesogastric, urogastric, cardiac and intestinal regions covered with rugosities and distinct granules; suborbital region with small granules on lateral parts; pterygostomial, subhepatic and sub-branchial regions rugose to granulose (Fig. 2A, D, F). Epigastric cristae distinct, rugose, not cristate, separated by broad, median Y-shaped furrow; epigastric cristae just anterior of postorbital cristae, separated by short furrow; postorbital cristae distinct, margin uneven, prominently raised, subparallel to frontal margin, outer edge relatively low, not prominent (Fig. 2A, D, F). Cervical grooves deep, not reaching lateral margins, connected to deep H-shaped median gastric groove (Fig. 2A, F). Frontal margin almost straight, appears entire in dorsal view, gently sinuous in frontal view (Fig. 2A, D, F). External orbital tooth distinct, triangular, outer margin more than twice length of inner margin, demarcated from rest of anterolateral margin by deep V-shaped cleft; epibranchial tooth prominent, sharp (Fig. 2A, F). Anterolateral margins convex, cristate, lined with sharp granules, appears serrated (Fig. 2A, F). Posterolateral margin gently sinuous, converging towards convex posterior carapace margin (Fig. 2A, F). Orbits subovate; eye filling orbital space; eye peduncle relatively short, stout; cornea large, round, pigmented (Fig. 2D). Supraorbital margin almost straight (Fig. 2F). Suborbital margin concave, complete, lined with low granules (Fig. 2D). Antennae short, stretching across base of eyes; antennules short, folding transversely in rectangular fossa (Fig. 2D). Posterior margin of epistome with distinct median triangle, lateral margin sinuous (Fig. 2D).

Third maxillipeds covering most of buccal cavity when closed; ischium subrectangular, with distinct median groove, surface with scattered pits and short setae; merus subquadrate, slightly wider than long, surface rugose, margins cristate, anteroexternal angle angular but not produced; exopod slender, reaching to about one-third length of merus, with elongate flagellum that reaches across width of merus (Fig. 3F).

Chelipeds asymmetrical, right larger (Fig. 2A). Anterior margin of basis-ischium lined with small sharp granules; margins of merus lined with low sharp granules, appears weakly serrated. Outer surface of carpus rugose, inner distal angle with large sharp tooth and basal tooth (Fig. 2A). Outer surfaces of chelae strongly rugose, upper part rugose with granules; major chela stouter, shorter than minor chela (Fig. 3H, I). Fingers of major chela short, stout, gently curved, subequal to palm, outer surface lined with 3 rows of pits; cutting edges of both fingers with variously sized sharp teeth and denticles; dorsal margin of dactylus with low tubercles and granules (Figs 2A, 3H). Fingers of minor chela similar to major chela in form but relatively more slender (Figs 2A, 3I).

Ambulatory legs short, segments relatively stout; second pair longest, last pair shortest (Fig. 2A). Merus short, stout, outer surface rugose, dorsal margin uneven, subcristate, without subdistal spine or tooth; carpus rugose, dorsal margin with crista, outer surface with low submedian crista on first to third legs, that on fourth leg smooth; dorsal margin of propodus with crista, outer surface with low, submedian crista; dactylus relatively short, gently curved, quadrate in cross section, margins with short, sharp pectinate spines (Fig. 2A).

Thoracic sternum, notably sternites 3 and 4, relatively broad, surface with pits and scattered short, stiff setae (Fig. 3A). Sternites 1, 2 completely fused to form broadly triangular plate; separated from sternite 3 by distinct, gently concave suture (towards buccal cavity); sternites 3, 4 completely fused, with shallow incomplete groove demarcating suture (Fig. 3A, B, E). Penis coxal, on condyle of coxa of fourth ambulatory leg. Sternopleonal cavity deep, reaching imaginary line connecting posterior edges of cheliped coxae (Fig. 3A, E). Male pleonal locking tubercle low, round, on posterior third of sternite 5 (Fig. 3E).

Pleon triangular, all somites and telson free; telson triangular, lateral margins gently sinuous; somite 6 transversely rectangular, much wider than long, lateral margin gently convex; somites 3–5 trapezoidal, gradually decreasing in width, increasing in length; somites 1 and 2 subrectangular, very wide, reaching to bases of coxae of fourth ambulatory legs, thoracic sternite 8 not visible when pleon closed (Fig. 3A, B).

G1 relatively stout; subterminal segment gently sinuous, proximal part broad, gradually tapering to median part, outer margin with distinct broad cleft on distal part; clearly separated from terminal segment by prominent dilation; terminal segment subcylindrical, no dorsal flap visible, gently curving outwards, distal part tapering to rounded tip (Fig. 4A–D). G2 elongate, much longer than G1; basal segment longer than distal segment (Fig. 4E).

##### Variation.

**Figure 5.**
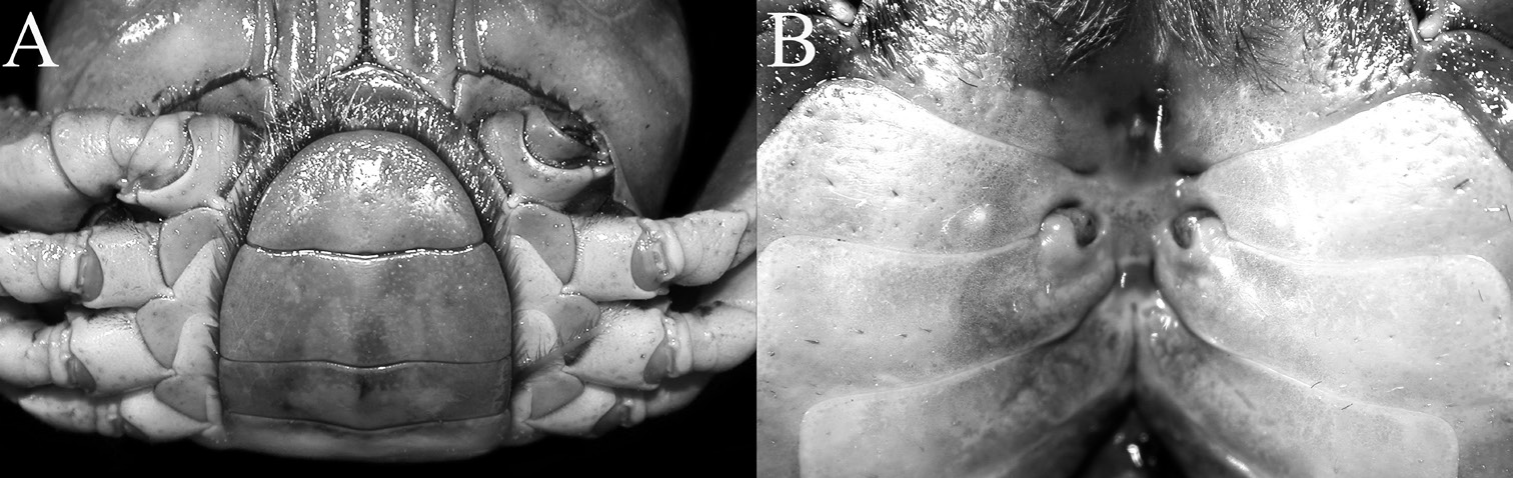
*Indochinamonkhinpyae* sp. n., paratype female (39.3×31.1 mm) (ZRC 2018.0714). **A** anterior thoracic sternum and pleon **B** sternopleonal cavity and vulvae.

The carapace tends to get less broad in smaller specimens and females (width to length ratio 1.26–1.30). The regions in smaller specimens is less sculptured (Fig. 2B, E) with the rugosities restricted mostly to lateral margins (Fig. 2C, G). The third maxilliped ischium is slightly longer in smaller individuals (Fig. 3G). The male pleon is proportionately less broad in smaller individuals with somite 6 more quadrate as they are smaller (Fig. 3C, D). In adult males, the G1 does not vary substantially although the cleft on the outer part of the distal section of the G1 subterminal segment is relatively less distinct (Fig. 4F). Smaller males (ca. 30 mm carapace width), however, not only have the G1 terminal segment relatively shorter and less curved, the cleft on the subterminal segment is also not discernible (Fig. 4H). The adult female has the pleon completely covering the thoracic sternum (Fig. 5A), the vulva is large, raised, ovate and positioned on the anterior half of sternite 6, pushing into the margin with sternite 5 (Fig. 5B).

##### Etymology.

The species is named after Ms Khin Pyae Pyae Thaw Thar who collected the specimens used for this study. Her name is used here as a noun in apposition.

##### Colour.

In life, the dorsal surfaces of the carapace and outer surfaces of the chelipeds are dark brown; with the ventral surfaces orangish-red; and the ambulatory legs are dark brown to orangish red (Fig. 1).

##### Habitat.

The type locality, Malikha, is a fast-flowing river, the substrate consisting of rocks of various sizes, with the bank sandy. The banks are densely lined with tall trees. This river is a branch of the Ayeyarwady River (= Ayrwarwady River or Myitsone) and is about 43 km north of Myitkyina, the capital city of Kachin State.

##### Remarks.

Five species of *Indochinamon* have been reported from and near Myanmar: *I.andersonianum*, *I.edwardsii*, *I.hirtum*, *I.hispidum* and *I.tritum* (cf. Alcock 1909, 1910; Bott 1970; Yeo and Ng 2007). All these species were collected by John Anderson from the area east of Bhamo, mostly in the Kakhyen Hills (= Kachin Mountains), in what is today Myanmar and Yunnan (China). One site, Ponsee, which is the type locality of *I.edwardsii* and *I.hispidum* (and where *I.andersonianum* has also been found), does not appear in most modern maps but this village is in the Dehong, Longchuan area in Yunnan, China (ca. 24°25'34.5"N, 97°53'57.3"E) (cf. Anderson 1876). Until the present record of *I.khinpyae*, no species had been reported from the mountains north of Bhamo and Myitkyina in Myanmar.

Adult male specimens of *I.khinpyae* have a strongly sculptured and very rough carapace (Fig. 2F), the G1 terminal segment is relatively long, gently curved, distally bent and the dorsal margin has no trace of a flap (Fig. 4A–D, F, H). In *I.andersonianum*, even large males (50 mm carapace width) have the gastric regions relatively smooth with the rest of the surfaces also less rugose and granulose, and the male pleon is proportionately more narrow (Wood-Mason 1871: pl. 27 figs 16, 17, 20; Bott 1970: pl. 44 fig. 14; unpublished data). The G1 of *I.andersonianum* is also quite different with the terminal segment straight, slender and tapering towards the tip (Bott 1970: pl. 37 fig. 16). The taxonomy of *I.andersonianum* has been confused and many species previously referred to it have turned out to be other taxa (see Ng and Naiyanetr 1993). The figure of *I.andersonianum* by Alcock (1910: pl. 10 fig. 40) is actually a separate species, *Potamiscusrangoonensis* (Rathbun, 1904) (unpublished data). The G1 of the smaller paratype male of *I.khinpyae* (34.3×26.6 mm, ZRC 2018.0714) superficially resembles that of *I.edwardsii* (the type of which is about the same size) but in *I.edwardsii*, the anterolateral margins are prominently serrated even in smaller specimens (Wood-Mason 1871: pl. 27 figs 11, 12; Alcock 1910: pl. 14 fig. 43; unpublished data) (versus anterolateral margins finely granulated or weakly serrated in *I.khinpyae*; Fig. 2F, G); the upper part of the palm of the chela has many large tubercles (Wood-Mason 1871: pl. 27 figs 11, 14; Alcock 1910: pl. 14 fig. 43; unpublished data) (versus with no large tubercles present in *I.khinpyae*; Figs 2A–C, 3H–I); and the lateral margins of the male telson are concave (Wood-Mason 1871: pl. 27 fig. 14; unpublished data) (versus lateral margins gently sinuous to almost straight in *I.khinpyae*; Fig. 3A, C, D). These differences also apply for *I.hirtum* (cf. Alcock 1919: pl. 10 fig. 42; unpublished data). Compared to *I.tritum*, known only from a 35.8×27.4 mm female from the Shitee Hills in the Kakhyen Hills in Yunnan (just north of Ponsee, cf. Anderson 1876: 420), the lateral margin of the postorbital crista of *I.khinpyae* is less clearly marked (Fig. 2F, G) (versus distinctly formed and clearly demarcated from the lateral branchial region in *I.tritum*; cf. Alcock 1910: pl. 14 fig. 69); and the propodus of the last ambulatory leg is proportionately longer (Fig. 2A–C) (versus much shorter in *I.tritum*; cf. Alcock 1910: pl. 14 fig. 69). Compared to *I.hispidum*, described from a male 43.0×31.0 mm from Ponsee, *I.khinpyae* can easily be distinguished by its more rugose dorsal carapace surface (Fig. 2A, B, F) (versus smooth dorsal carapace surface in *I.hispidum*; cf. Wood-Mason 1871: pl. 27 figs 1, 2); rugose outer surface of the chela (Fig. 3H, I) (versus smooth in *I.hispidum*; cf. Wood-Mason 1871: pl. 27 fig. 4); and the male pleon is proportionately broader with the telson more broadly triangular (Fig. 3A, C, D) (versus male pleon more narrow with the telson acutely triangular in *I.hispidum*; cf. Wood-Mason 1871: pl. 27 fig. 5).

With regards to the other species of *Indochinamon*, they can be separated into several groups on the basis of their G1s. The type species, *I.villosum*, has a relatively short and stout G1 terminal segment which is gently bent and is conical to subconical in shape without an obvious dorsal flap, a character shared with *I.ahkense*, *I.bavi*, *I.bhumibol*, *I.boshanense*, *I.changpoense*, *I.chinghungense*, *I.dangi*, *I.daweishanense*, *I.flexum*, *I.guttum*, *I.jianchuan*, *I.jinpingense*, *I.kimboiense*, *I.menglaense*, *I.mieni*, *I.orleansi*, *I.ou*, *I.parpidum*, *I.phongnha*, *I.tannanti*, *I.xinpingense*, and *I.yunlongense* (including *I.edwardsii* and *I.hispidum*) (cf. Bott 1970; Yeo and Ng 1998; Dai 1999; Naruse et al. 2011, 2018; unpublished data). The other species have G1 terminal segments which are slender, elongate, and straight or curved; or relatively short and strongly bent (cf. Ng and Naiyanetr 1993; Dai 1999; Do et al. 2016; Naruse et al. 2018; unpublished data). The G1 of *I.khinpyae* closely resembles that of *I.changpoense* and *I.daweishanense* (both from Yunnan) but these two species have only a shallow cleft on the outer margin of the subdistal part of the subterminal segment, and the terminal segment is proportionately shorter and straighter (Dai 1999: figs 85–4, 5; 87–4, 5), even though the types are comparable in size to the holotype of *I.khinpyae*. Similarly, *I.yunlongense* (described from a small male 19.0×16.1 mm from Yunnan) has a superficially similar G1 structure to *I.khinpyae*, except that the terminal segment is much straighter (Dai 1999: fig. 84–4, 5). The strongly sculptured and rugose carapace of large *I.khinpyae* allies it with large species like *I.kimboense* and *I.bavi* (both from Vietnam) but in these species, the cleft on the outer margin with of the G1 subterminal segment is shallow and not distinct (cf. Naruse et al. 2011: fig. 3a, b, d, e), even for specimens larger than the holotype of *I.khinpyae*, which has a prominent broad cleft (Fig. 4A–D). The G1 terminal segment of *I.kimboense* and *I.bavi* (as well as *I.cua*, *I.orleansi*, *I.ou* and *I.tannanti*) are also distinctly tapering towards the tip (Naruse et al. 2011: fig. 3a, b, d, e), unlike the subtruncate condition in *I.khinpyae* (Fig. 4A–D). Compared to *I.phongnha* (from Vietnam), which also has the carapace regions distinct, the surfaces are smoother, notably the median and posterior parts which are smooth, even in large specimens (Naruse et al. 2011: fig. 7) (strongly rugose in large *I.khinpyae*; Figs 1A, 2A, F); and the G1, while it has a strong cleft on the outer margin of the subterminal segment, the terminal segment is sharply tapering (Naruse et al. 2011: fig. 9a, b) (terminal segment subcylindrical in large *I.khinpyae*; Fig. 4A–D). The strong cleft on the outer margin of the G1 subterminal segment of *I.khinpyae* is character also shared with *I.cua* from Thailand, but in this species, the cleft is relatively broader and the terminal segment is tapering distally (Yeo and Ng 1998: fig. 4B, C, E, G); and the carapace regions are proportionately much smoother (Yeo and Ng 1998: fig. 7A). In *I.lipkei* from Thailand, the dorsal carapace surface, even in large specimens, is less well marked with the median parts much less rugose (Ng and Naiyanetr 1993: fig. 12A); pleonal somite 6 is distinctly trapezoidal in shape (Ng and Naiyanetr 1993: fig. 12C); and the G1 terminal segment is strongly bent at about 60° along the longitudinal axis (Ng and Naiyanetr 1993: fig. 47B–E) (versus the dorsal carapace regions are more rugose, pleonal somite 6 is weakly trapezoidal, and the G1 terminal segment is bent at about 45° in *I.khinpyae*; Figs 2F, 3A, C, D, 4A–D).

*Indochinamonkhinpyae* is not known to be threatened by any developments, and the forests and streams where it has been found are isolated and not easily assessible by man. As such, the species is classified under taxa of Least Concern for the moment (cf. Cumberlidge et al. 2009, 2012).

A note on *I.manipurense* (Alcock, 1909) is necessary. Takeda et al. (2012: 207) noted that specimens they had of this species did not possess a flagellum on the exopod of the third maxilliped, and as such, the species should be transferred to *Potamiscus* Alcock, 1909. However, the types of this species do have a flagellum (Yeo and Ng 2007; unpublished data), so Takeda et al.’s (2012) specimens will need to be checked to ascertain their identity. As such, for the moment, we retain the species in the genus *Indochinamon*.

## Supplementary Material

XML Treatment for Indochinamon

XML Treatment for Indochinamon khinpyae
